# Impact of Grapefruit Consumption on Plasma Concentrations of Psychiatric Medications through CYP3A4 Inhibition

**DOI:** 10.1192/j.eurpsy.2025.756

**Published:** 2025-08-26

**Authors:** D. B. Valle, D. J. Cox, B. R. Carr

**Affiliations:** 1 College of Medicine; 2 Department of Psychiatry, University of Florida, Gainesville, United States

## Abstract

**Introduction:**

The interaction between grapefruit juice and certain psychiatric medications can lead to significant clinical implications due to the inhibition of the cytochrome P450 3A4 (CYP3A4) enzyme (Fuhr et al. CPT 2023, 114(2), 266-275; Guttman et al. Phytother. Res 2020, 34(5), 1168-1176; Paine et al. Drug Metab. Dispos 2004, 32(10), 1146-1153; Paine et al. J. Pharmacol. Exp. Ther 2005, 312(3), 1151-1160; Schmiedlin-Ren et al. Pharmacol. Ther 1997, 66(2), 234-241). Grapefruit juice contains furanocoumarins, specifically bergamottin and 6’,7’-dihydroxybergamottin (DHB), which irreversibly inhibit CYP3A4, potentially increasing drug plasma concentrations and the risk of adverse effects (Bailey et al. CPT 1998, 64(3) 248-256; de Castro et al. J. Agric. Food Chem 2006, 54(7) 2498-2503; Row et al. J. Med. Chem 2005, 49(20), 6139-6146).

**Objectives:**

This review aims to quantify the impact of grapefruit juice on the plasma concentrations of buspirone, carbamazepine, and diazepam and to understand the duration of these effects to better manage patient safety.

**Methods:**

A comprehensive review of existing pharmacokinetic studies (Furukori et al. BJCP 2003, 55(3), 307-311; Lane et al. Psychopharm 2001, 155(3), 356-359; Tanaka et al. Clin. Pharm 2013, 52(5), 397-420; Wang et al. CPT 1993, 65(3), 314-321; Yasui et al. Psychopharm, 145(1), 84-87) was conducted to gather data on the effects of grapefruit juice on CYP3A4 substrate psychiatric medications. Quantitative increases in plasma concentration metrics (AUC and Cmax) were extracted, and the duration of the inhibition effect was analyzed.

**Results:**

Buspirone plasma concentrations increased by 4.3-fold, with effects lasting 24 hours. Carbamazepine showed a 1.4-fold increase in AUC and a 1.2-fold increase in Cmax, with effects persisting up to 24 hours. Diazepam concentrations increased by 3-fold in AUC and 2-fold in Cmax, with an effect duration of 24 hours. No significant interaction was observed for clozapine and haloperidol.

**Conclusions:**

Grapefruit juice significantly increases the plasma concentrations of buspirone, carbamazepine, and diazepam by inhibiting CYP3A4, with effects lasting up to 24 hours. Clinicians should educate patients on avoiding grapefruit consumption while on these medications and monitor for potential toxicity. Further research is needed to develop guidelines for managing these interactions and to explore genetic variations in response to grapefruit consumption.

**Disclosure of Interest:**

None Declared

